# Changes in Growth Performance and Ileal Microbiota Composition by Xylanase Supplementation in Broilers Fed Wheat-Based Diets

**DOI:** 10.3389/fmicb.2021.706396

**Published:** 2021-07-15

**Authors:** Jian Wang, Sujie Liu, Jiayu Ma, Xiangshu Piao

**Affiliations:** State Key Laboratory of Animal Nutrition, College of Animal Science and Technology, China Agricultural University, Beijing, China

**Keywords:** xylanase, wheat, broiler, ileal, microbiota

## Abstract

Xylanase exerts key roles in improving growth performance and intestinal health of broilers fed wheat-based diets. However, knowledge is limited regarding effects of xylanase supplementation on ileal microbiota in broilers. A total of 128 one-day-old broilers (initial BW 48.03 ± 0.33 g) were selected to investigate effects of xylanase (AT-xynA) on growth performance, ileal morphology, microbiota composition, immune response, antioxidant capacity, and endocrine peptide levels in broilers. Broilers were randomly allotted into two dietary treatments (*n* = 8), namely, a wheat-soybean basal diet and a basal diet with 4,000 U/kg AT-xynA (XY). On days 7, 14, 21, and 42, broilers were weighted and ileal tissues were sampled. Ileal digesta samples were collected for analyzing microbiota composition on days 21 and 42. The results showed that AT-xynA could improve average daily weight gain and average daily feed intake, and there were interactions between diet and age of broilers (*p* < 0.05). On days 21 and 42, xylanase supplementation decreased ileal microbiota α-diversity, and the relative abundance of potentially pathogenic microbiota, such as phylum Proteobacteria, family Moraxellaceae and Staphylococcaceae, genus *Staphylococcus*, *Pseudomonas*, *Streptococcus*, and *Enterococcus*, increased the abundance of *Lactobacillus* (*p* < 0.05). Moreover, the reduction in acetate concentration and abundance of short-chain fatty acid-producing bacteria was also observed in broilers from XY group (*p* < 0.05). AT-xynA increased ileal villus height, glucagon-like peptide-1, and insulin-like growth factor-1 concentrations and decreased interleukin-1β, interleukin-6, tumor necrosis factor-α, and malondialdehyde content in broilers, and these positive effects on intestinal health were greater in young broilers. In conclusion, xylanase supplementation to wheat-based diets could improve ileal intestinal morphology and immune function, and alleviate excess fermentation of bacteria, which may be related to changes of intestinal microbiota. In addition, the positive effects of xylanase on intestinal health were more pronounced in young broilers, thus contributing to subsequent improvement in growth performance of broilers.

## Introduction

Wheat can be included in poultry diets as a major energy source, especially in European broilers diets (Ball et al., 2013). However, the non-starch polysaccharides (NSP) present in wheat cell walls showed anti-nutritional effects, which might negatively affect broilers performance (Smeets et al., 2018). The increase of digesta viscosity in the small intestine due to soluble NSPs in wheat is a major reason for anti-nutritional effects, and the increase will reduce diffusion rate of substrates and digestive enzymes, inhibit effective interaction between them in the intestine, thus reduce digestion and absorption of nutrients, and result in poor performance (Choct et al., 1996; Bedford and Schulze, 1998). Moreover, negative effects of soluble NSPs on performance might be indirectly associated with microbiota composition in the small intestine, soluble NSPs lead to a slow digesta flow, decrease small intestine oxygen tension, and thereby promote the growth of anaerobic microorganisms, and the changes of microbiota composition could negatively affect physiological function of the intestine (Choct et al., 1996; Raza et al., 2019). Therefore, the application of NSP degrading enzymes in wheat-based diets is necessary to improve growth performance and intestinal health of broilers.

Arabinoxylans are the major NSPs in wheat, the content of arabinoxylan ranges from 4.1 to 9.0%, and thus, endo-β-1,4-xylanases are commonly supplemented in broilers wheat-based diets for degradation of arabinoxylans (Rosicka-Kaczmarek et al., 2016; Cardoso et al., 2018). It is generally believed that the mechanism of NSP-degrading enzymes in broilers includes reducing digesta viscosity, releasing encapsulated nutrients, and providing fermentable NSP hydrolysis products (Masey et al., 2014). At the present, positive effects of xylanase on growth performance of broilers have been well accepted; however, the effect of xylanase on intestinal health is still an active field of research (Choct et al., 1999; Cardoso et al., 2018). The xylanase is generally considered to work through two stages, namely, an ileal stage and a cecal stage (Engberg et al., 2004). Most studies focused on effects of xylanase on cecal microbiota; the knowledge regarding ileal microbiota was limited (McCafferty et al., 2019a,b; Petry et al., 2021). In fact, NSP-degrading enzyme improves small intestine digestion as a consequence decreases undigested substrates in the small intestine, which might also result in the reduction of fermentative microorganisms and potentially pathogenic microorganisms in the ileum (Danicke et al., 1999; Raza et al., 2019). At the present, effects of xylanase on ileal microbiota composition were just analyzed by conventional molecular ecology techniques; thus, changes of microbiota composition still need to be further explored (Hübener et al., 2002; Engberg et al., 2004). 16S rRNA gene sequencing has been proved to be a powerful tool for the assessment of diversity and composition of microbiota due to its informative and predictive potential. Moreover, there are interactions between ileal microbiota and immune responses, and the higher pathogen loads in the small intestine could also contribute to oxidative stress and inflammatory responses (Tiwari et al., 2018). Therefore, we performed an experiment to determine effects of xylanase on growth performance, intestinal morphology, immune response, antioxidant capacity, barrier function, and endocrine peptide levels of broilers. Subsequently, ileal microbiota was analyzed through high-throughput sequencing of the 16S rRNA gene, and correlations between microbiota composition with intestinal morphology and immunity function were evaluated.

## Materials and Methods

The Laboratory Animal Welfare and Animal Experimental Ethical Inspection Committee of China Agricultural University (Beijing, China) approved all the experimental protocols in this study (AW70601202-1-1).

### Preparation of Xylanases

The xylanase (AT-xynA) in this study was provided by the Ministry of Agriculture Feed Industry Center Lab in China Agricultural University (Beijing, China). The xylanase (AT-xynA) was prepared as previously described (Wang et al., 2020). One unit of activity is defined as the amount of xylanase that releases 1 μmol of xylose per min at the optimal conditions (pH 3.0 and 50°C).

### Experiment Animals, Management, Design, Diets, and Processing Procedure

The 128 one-day-old broilers (Arbor Acres Poultry Breeding Company, Beijing, China) with initial body weight 48.03 ± 0.33 g were randomly divided into two treatments, and each treatment consisted of eight replicates with eight broilers in each replicate. The two dietary treatments consisted of a wheat-soybean basal diet (CON) and a CON with 4,000 U/kg AT-xynA (XY). Experimental diets were supplied throughout two phases: a starter phase (days 0–21) and a grower-finisher phase (days 21–42). The ingredients of experimental diets were exhibited (Table 1), and all essential nutrients of the basal diet satisfied nutrient requirements of broilers following National Research Council (1994) recommendations. The management of broilers in this experiment followed the guidelines of raising AA broilers described by Mahfuz et al. (2019).

**Table 1:** Composition and nutrient levels of basal diets (%, as-fed basis).

Item	days 0–21	days 21–42
*Ingredients*		
Corn	33.16	37.45
Wheat	33.00	31.91
Soybean meal, 43%	23.05	19.22
Fish meal, 64.6%	3.62	3.74
Soybean oil	3.60	4.50
Dicalcium phosphate	1.17	1.09
Limestone	1.24	1.05
Salt	0.30	0.30
L-lysine HCl, 78%	0.12	0.11
DL-methionine, 98%	0.16	0.05
L-threonine, 98%	0.08	0.08
Vitamin-mineral premix^a^	0.50	0.50
Total	100.00	100.00
*Nutrient content*^b^		
Metabolizable energy, kcal/kg	3,050	3,150
Crude protein	20.53	18.95
Calcium	1.00	0.90
Total phosphorous	0.68	0.65
Lysine	1.10	1.00
Methionine	0.50	0.38

a Vitamin A, 11,000 IU; vitamin D, 3,025 IU; vitamin E, 22 mg; vitamin K_3_, 2.2 mg; thiamine, 1.65 mg; riboflavin, 6.6 mg; pyridoxine, 3.3 mg; cobalamin, 17.6 μg; nicotinic acid, 22 mg; pantothenic acid, 13.2 mg; folic acid, 0.33 mg; biotin, 88 μg; choline chloride, 500 mg; iron, 48 mg; zinc, 96.6 mg; manganese, 101.76 mg; copper, 10 mg; selenium, 0.05 mg; iodine, 0.96 mg; and cobalt, 0.3 mg.

b Values are calculated according to NRC.

### Growth Performance, Sample Collection, and Intestinal Morphology Analysis

Body weight and feed intake were recorded on days 0, 7, 14, 21, and 42 to calculate average daily weight gain (ADG), average daily feed intake (ADFI), and feed conversion ratio (FCR) of broilers. On days 7, 14, 21, and 42, one broiler from each replicate was slaughtered for intestinal sample collection, and middle parts of ileum were collected and then immediately placed in 10% buffered formalin. The formalin-fixed tissues were embedded in paraffin and cut into about 5 mm slices. Then, samples were dyed with hematoxylin and eosin (HE) for histological examination, and six well-oriented crypt and villi from each slice were selected for determination of the villi height (VH) and crypt depth (CD) through a light microscope. Ileal tissue samples of slaughtered broilers from each treatment were also collected on days 7, 14, 21, and 42 and then maintained at −80°C. On days 21 and d 42, broilers ileal digesta from each treatment were sampled to analyze composition and metabolites of the intestinal microbiota.

### Microbiota Profiling

The extraction for total bacterial genomic DNA of ileal digesta samples was performed using the EZNA Stool DNA kit (Omega Bio-Tek, Norcross, GA, United States) following instructions of manufactures. The PCR conditions for amplification of the V3–V4 region of the bacterial 16S rRNA gene were as follows: 3 min at 95°C, 27 cycles of 95°C for 30 s, 55°C for 30 s, 72°C for 45 s, and 10 min at 72°C. The 16S rRNA gene was amplified with primers 338F (5'barcodeACTCCTAC GGGAGGCAGCAG3') and 806R (5'GGACTACHVGGGTWTCTAAT3'). The 2% agarose gel-electrophoresis was used for the detection of purity of PCR products, and the products were purified with the AxyPrep DNA Gel Extraction kit (Axygen Biosciences, Union City, CA, United States). Purified amplicons were quantified by QuantiFluor (Promega, Madison, WI) and then paired-end sequenced (2 × 250) on the Illumina MiSeq platform (Illumina, San Diego, CA, United States). Raw fastq files were de-multiplexed and quality-filtered with QIIME (version 1.70). Operational taxonomic units (OTUs) were clustered using a 97% similarity cut-off with UPARSE (version 7.1), and chimeric sequences were removed using UCHIME. Representative sequence of OTUs was obtained based on Ribosomal Database Project classifier. The sequence data were uploaded to the NCBI Sequence Read Archive database (accession number: PRJNA725811).

### Microbial Metabolites

As reported by previous study, short chain fatty acid (SCFA) concentrations in ileal digesta were detected by using an ion chromatographic method (Liu et al., 2017). About 0.5 g of ileal digesta sample was mixed with 8 ml of distilled water, and after 30 min of ultrasonic treatment, the mixture was centrifuged at 8,000 × g for 10 min to achieve the supernatant. We filtered the diluted supernatant using a 0.22 μm filter and finally injected the supernatant into a high-performance ion chromatograph (ICS 3000 Dionex, United States). SCFAs were separated by an AG11 guard column and an AS11 analytical column under the gradient condition (0–5 min, 0.8–1.5 mm; 5–10 min, 1.5–2.5 mm; and 10–15 min, 2.5 mm); the flow rate was 1.0 ml/min. The gradient was carried out with potassium hydroxide.

### Tight Junction Gene Expression

Total RNA of ileal tissues was isolated by using mirVana Kit (Ambion) following the manufacturer’s directions. Then, RNA yield was measured by a NanoDrop 2000 spectrophotometer (Thermo Fisher Scientific, Waltham, MA, United States), and RNA quality was assessed by 1% agarose gels electrophoresis. PrimeScript RT reagent kit (TaKaRa, China) was used to complete the reverse transcription following the manufacture’s recommendations. Real-time PCR was performed with LightCycler^®^ 480 II Real-time PCR Instrument (Roche, Swiss), and the mixture consisted of 1 μl of cDNA, 5 μl of 2 × PerfectStart^™^ Green qPCR SuperMix, 0.2 μl of forward primer, 0.2 μl of reverse primer, and 3.6 μl of nuclease-free water. The reaction conditions were 30 s at 94°C, 45 cycles of 5 s at 94°C, and 30 s at 60°C. After the qPCR run, the expected PCR product was recognized by analyzing melting curve. The primers for genes associated with intestinal barrier function including zonula occludens-1 (*ZO-1*; forward primer: 5'AGATGGACAGCATCAACG3'; reverse primer: 5'CTGCCACATCCTG GTATT3'), occludin (forward primer: 5'TGGTACTGACCAACGTAGTTC3'; reverse primer: 5'AGGAGTGACATCTAATAAAGCG3'), and housekeeping genes [β-actin and glyceraldehyde phosphate dehydrogenase (GAPDH); forward primer: 5'GAAGGCTGGGGCTCATCTG3'; reverse primer: 5'CAGTTGGTGGTGCACGA TG3'] were synthesized by TsingKe Biotech (Beijing, China). The relative expression of *ZO-1* and occludin gene was analyzed by the 2^−ΔΔCt^ method and normalized to reference gene (GAPDH) (Livak and Schmittgen, 2001).

### Intestinal Antioxidant and Immune Function

The malondialdehyde (MDA) concentration and the activity of superoxide dismutase (SOD) in the ileum were measured using commercial assay kits (Nanjing Jiancheng Bioengineering Institute, Nanjing, China) following the manufacturer’s instructions. The levels of interleukin-1β (IL-1β), interleukin-6 (IL-6), interleukin-10 (IL-10), tumor necrosis factor-α (TNF-α), and interferon-γ (IFN-γ) in the ileum were determined by enzyme-linked immunosorbent assay (ELISA) kits (Nanjing Jiancheng Bioengineering Institute, Nanjing, China).

### Endocrine Peptides

The determination of insulin-like growth factor 1 (IGF-1), epidermal growth factor (EGF), glucagon-like peptide-1 (GLP-1), and glucagon-like peptide-2 (GLP-2) levels in the ileum was detected with the ELISA kits (Laibo Tairui Technology Development Co., Ltd., Beijing, China) according to manufacturer’s instructions.

### Statistical Analysis

The data analysis was performed by using the procedure of SAS (version 9.2, 2008), and results were expressed as means ± standard error of the mean (SEM). When evaluating growth performance, the replicate was considered as an experimental unit. For other parameters (intestinal morphology, tight junction gene expression, antioxidant activities, inflammation function, endocrine peptide levels, and SCFA concentrations), the individual slaughtered broiler was treated as an experimental unit. The student’s test procedure was performed to compare two groups: *p* < 0.05 was found to be statistically significant, whereas 0.05 ≤ *p* ≤ 0.10 was regarded as a significance of trend. The α-diversity indices, including Shannon diversity index (Shannon), ACE estimator (Ace), Chao 1 estimator (Chao), observed richness (Sobs), Good’s coverage (Coverage), and phylogenetic diversity (PD), were calculated by using QIIME. The procedure of the linear discriminant analysis (LDA) effect size linear discriminant analysis (LEfSe) algorithm was used to find differences in the relative abundance of bacteria between two treatments with LDA score of bacteria exceeding 2.0. We used R package of “Hmisc” to calculate the Spearman’s correlation coefficient. Functions of bacterial community from ileal digesta samples were predicted by PICRUSt analysis.

## Results

### Growth Performance

As given in Table 2, ADG, ADFI, and FCR of broilers were significantly affected by age (*p* < 0.01). The addition of AT-xynA could significantly improve ADG (*p* < 0.01) and ADFI (*p* = 0.030) of broilers, and tended to improve FCR (*p* = 0.056). Broilers from XY group showed higher ADG than those from CON group from days 14–21 and days 21–42 (*p* < 0.05). Compared with broilers fed with no AT-xynA, broilers fed diets containing AT-xynA also exhibited higher ADFI during days 21–42 and lower FCR during the third week (*p* < 0.05). For ADG (*p* < 0.01) and ADFI (*p* = 0.014), there were significant interactions between diet and age of broilers.

**Table 2:** Effects of AT-xynA on growth performance of broilers.^1^

Items	days 0–7		days 7–14		days 14–21		days 21–42		SEM	Value of *p*		
	CON	XY	CON	XY	CON	XY	CON	XY		Diet	Age	Diet*Age
Average daily gain, g/d	14.12^f^	14.55^f^	20.53^e^	21.62^e^	37.28^d^	40.34^c^	51.57^b^	60.10^a^	0.96	<0.01	<0.01	<0.01
Average daily feed intake, g	15.00^e^	15.18^e^	22.28^d^	23.30^d^	56.18^c^	56.38^c^	89.61^b^	98.89^a^	1.57	0.030	<0.01	0.014
Feed conversion ratio	1.06^d^	1.04^d^	1.09^d^	1.08^d^	1.52^b^	1.40^c^	1.65^a^	1.74^a^	0.04	0.056	<0.01	0.482

1 CON (*n* = 8) is AT-xynA un-supplemented group; XY (*n* = 8) is 4,000 U/kg AT-xynA supplemented group.

a-f Means with a row with no common superscript differ significantly.

### Ileal Morphology

**Figure 1:**
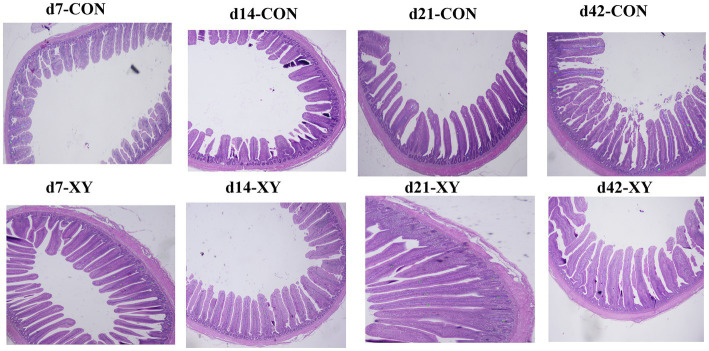
Representative histological micrographs of ileum in broilers. CON is AT-xynA un-supplemented group; XY is 4,000 U/kg AT-xynA supplemented group.

The light micrographs of ileal morphology are displayed in Figure 1, and data about the ileal morphology are given in Table 3. Compared with CON group, VH of ileum was greater in broilers from XY group on day 7 (*p* = 0.015) and day 21 (*p* = 0.010), and VH of ileum also showed a trend to increase in broilers from XY group (*p* = 0.097) on day 14. However, no differences were shown in CD and VH/CD values between two treatments.

**Table 3:** Effects of AT-xynA on ileal morphology of broilers.^1^

Items	CON	XY	Value of *p*
*day 7*			
Villus height, μm	293.40 ± 13.80	359.20 ± 13.94	0.015
Crypt depth, μm	64.26 ± 7.38	71.86 ± 11.19	0.592
Villus height to crypt depth ratio	4.72 ± 0.51	5.37 ± 0.79	0.513
*day 14*			
Villus height, μm	428.80 ± 18.56	688.00 ± 12.02	0.097
Crypt depth, μm	85.82 ± 7.75	116.40 ± 14.77	0.104
Villus height to crypt depth ratio	5.12 ± 0.39	5.79 ± 0.51	0.331
*day 21*			
Villus height, μm	492.70 ± 16.23	586.00 ± 31.51	0.010
Crypt depth, μm	95.98 ± 7.42	113.70 ± 1.25	0.136
Villus height to crypt depth ratio	5.21 ± 0.45	5.16 ± 0.21	0.930
*day 42*			
Villus height, μm	792.40 ± 19.21	838.90 ± 28.67	0.214
Crypt depth, μm	113.90 ± 7.15	110.10 ± 5.34	0.680
Villus height to crypt depth ratio	7.08 ± 0.52	7.73 ± 0.59	0.437

1 CON is AT-xynA un-supplemented group; XY is 4,000 U/kg AT-xynA supplemented group.

Values are expressed as means ± standard error of the mean (SEM), *n* = 6.

### Microbial Diversity and Community in Ileal Digesta

**Figure 2:**
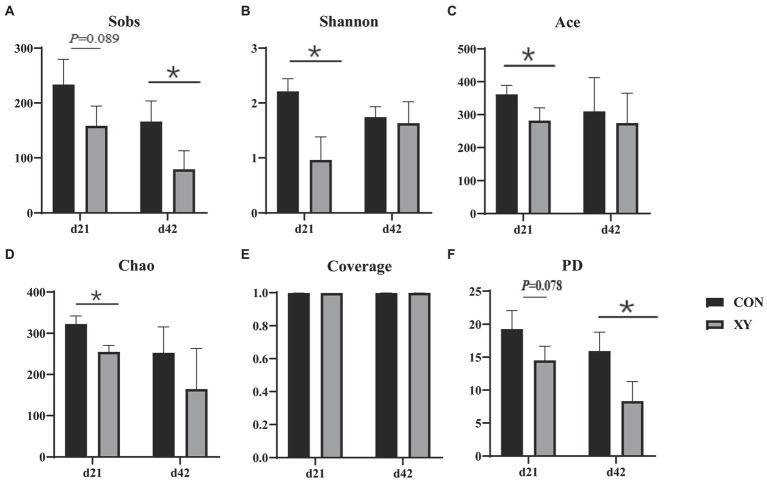
Effects of AT-xynA on alpha diversity of ileal microbiota in broilers. **(A)** Observed richness (Sobs), **(B)** Shannon diversity index (Shannon), **(C)** ACE estimator (Ace) **(D)** Chao 1 estimator (Chao) **(E)** Good’s coverage (Coverage), and **(F)** phylogenetic diversity (PD) of ileal microbiota in broilers on days 21 and 42. CON is AT-xynA un-supplemented group; XY is 4,000 U/kg AT-xynA supplemented group. The “*” indicates a significant difference between the two treatments (*p* < 0.05).

As shown in Figure 2, the α-diversity analysis, Shannon (*p* = 0.011), Ace (*p* = 0.044), and Chao (*p* = 0.009) indices were significantly lower in the ileum of broilers fed with AT-xynA, Sobs (*p* = 0.089) and PD (*p* = 0.078) indices showed trends to decrease in broilers from XY group on day 21. On day 42, broilers from XY group also showed lower Sobs (*p* = 0.041) and PD (*p* = 0.033) indices compared with broilers from CON group.

**Figure 3:**
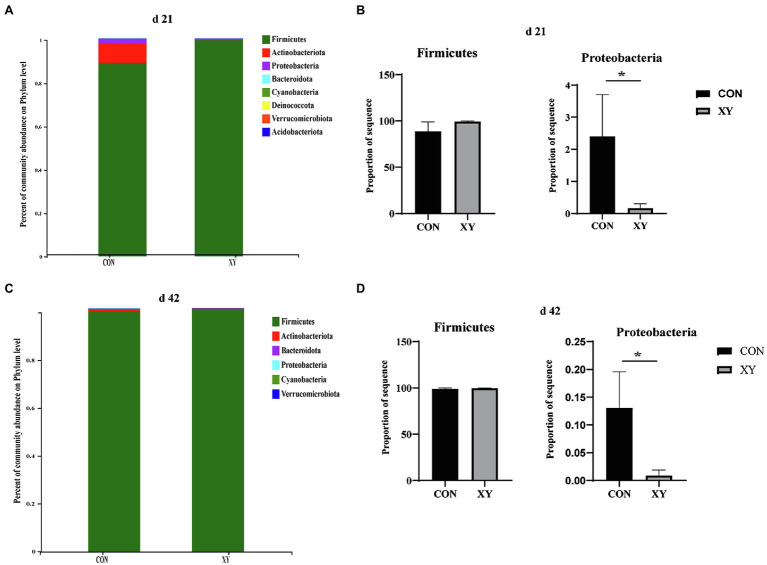
Effects of AT-xynA on ileal microbiota composition at the phylum level. **(A,C)** Ileal microbiota composition at the phylum level in broilers; **(B,D)** alterations of the abundance of bacterial phyla found in the ileum of broilers. CON is AT-xynA un-supplemented group; XY is 4,000 U/kg AT-xynA supplemented group. The “*” indicates a significant difference between the two treatments (*p* < 0.05).

We assessed the ileal microbiota at various taxonomic levels to evaluate differences in the microbiota composition induced by addition of AT-xynA. At the phylum level, the dominant bacterial group in CON group was Firmicutes, Actinobacteriota, Proteobacteria, Bacteroidota, Cyanobacteria, Deinococcota, Verrucomicrobiora, and Acidobacteria, and Firmicutes, Actinobacteriota, Proteobacteria, Bacteroidota, Cyanobacteria, and Verrucomicrobiora were the major phyla in XY group on day 21. In addition, on day 42, ileal microbiota was dominated by Firmicutes, Actinobacteriota, Proteobacteria, Bacteroidota, Cyanobacteria, and Verrucomicrobiora in CON group, and the major phyla in ileal microbiota of broilers from XY group were Firmicutes, Actinobacteriota, Proteobacteria, and Bacteroidota. As shown in Figure 3, the abundance of Proteobacteria significantly decreased in XY group compared with that in CON on day 21 (*p* = 0.042) and day 42 (*p* = 0.033). In contrast, the abundance of Firmicutes in XY group was higher than broilers fed diets un-supplemented AT-xynA on day 21 and day 42 but no significant differences were observed (day 21, CON: 88.82% vs. XY: 99.25%; day 42, CON: 98.89% vs. XY: 99.84%).

**Figure 4:**
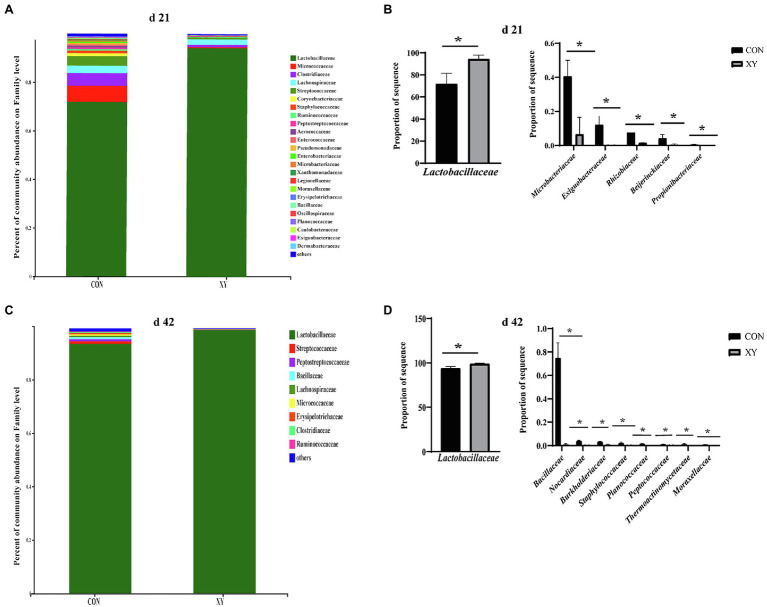
Effects of AT-xynA on ileal microbiota composition at the family level. **(A,C)** Ileal microbiota composition at the family level with the relative abundance higher than 0.1% in broilers; **(B,D)** alterations of the abundance of bacterial families found in the ileum of broilers. CON is AT-xynA un-supplemented group; XY is 4,000 U/kg AT-xynA supplemented group. The “*” indicates a significant difference between the two treatments (*p* < 0.05).

At the family level, the most dominant families (> 1%) in the ileum of broilers from CON group included Lactobacillaceae (71.83%), Micrococcaceae (6.66%), Clostridiaceae (5.23%), Lachnospiraceae (3.05%), Streptococcaceae (3.98%), Corynebacteriaceae (1.18%), and Staphylococcaceae (1.04%), whereas Lactobacillaceae (94.28%) and Lachnospiraceae (2.24%) were the most dominant families (> 1%) in XY group on day 21. On day 42, ileal microbiota was both dominated (> 1%) by Lactobacillaceae (CON: 94.23% vs. XY: 99.31%) in CON and XY groups. As shown in Figure 4, on day 21, the abundance of Lactobacillaceae (*p* = 0.019) was higher in ileal microbiota of broilers from XY group compared with those from CON group. In contrast, the abundance of Microbacteriaceae (*p* = 0.012), Exiguobacteraceae (*p* = 0.043), Rhizobiaceae (*p* = 0.040), Beijerinckiaceae (*p* = 0.045), and Propionibacteriaceae (*p* = 0.016) was higher in broilers from CON group on day 21. On day 42, the abundance of Lactobacillaceae also increased in ileal microbiota of broilers from XY group (*p* = 0.005), whereas the abundance of Bacillaceae (*p* < 0.01), Nocardiaceae (*p* < 0.01), Burkholderiaceae (*p* = 0.005), Staphylococcaceae (*p* = 0.013), Planococcaceae (*p* = 0.003), Peptococcaceae (*p* = 0.005), Thermoactinomycetaceae (*p* = 0.038), and Moraxellaceae (*p* = 0.003) was lower in ileal microbiota of broilers fed with AT-xynA.

**Figure 5:**
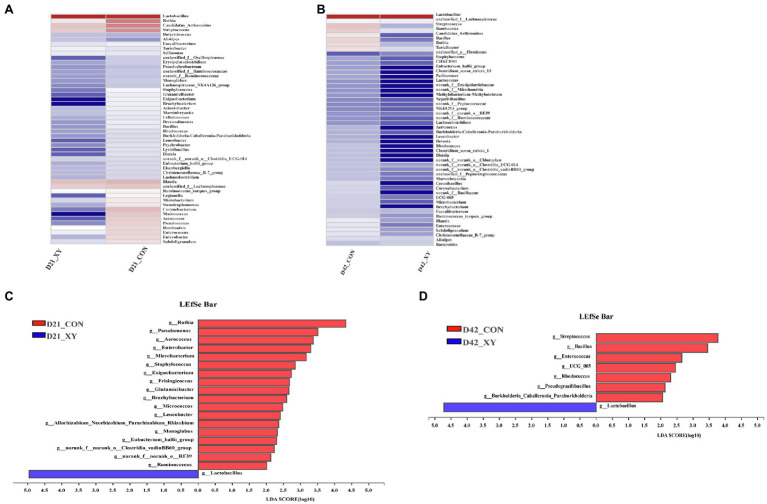
Effects of AT-xynA on ileal microbiota composition at the genus level. **(A,B)** Ileal bacterial community heatmap at the genus level; **(C,D)** the analysis of different bacteria communities at the genus level in the cladogram of Linear discriminant analysis effect size between two groups. CON is AT-xynA un-supplemented group; XY is 4,000 U/kg AT-xynA supplemented group.

At the genus level, as shown in Figure 5, the heatmap exhibited similarities and differences in bacterial community between two groups. In addition, differences in the composition of ileal microbiota at the genus level were further explored by the LEfSe method. The results indicated that compared with CON group, XY group was characterized by higher relative abundance of *Lactobacillus* (*p* < 0.05), lower abundance of *Rothia*, *Pseudomonas*, *Aerococcus*, *Enterobacter*, *Microbacteroium*, *Staphylococcus*, *Exiguobacterium*, *Frisingicoccus*, *Glutamicibacter*, *Brachybacterium*, *Micrococcus*, *Leucobacter*, *Allorhizobium_Neorhizobium_Pararhizobium_Rhizobiium*, *Monoglobus*, *Eubacterium_hallii_group*, *norank_f_norank_o_Clostridia_vadinBB60_group*, *norank_f_norank_o_RF39*, and *Ruminococcus* (*p* < 0.05) on day 21. Compared with microbiota composition at genus level on day 42, we also found that broilers from XY group had a higher proportion of *Lactobacillus* (*p* < 0.05) and lower proportion of *Streptococcus*, *Bacillus*, *Enterococcus*, *UCG_005*, *Rhodococcus*, *Pseudogracilibacillus*, and *Burkholderia_Caballeronia_Paraburkholderia* (*p* < 0.05) than CON group.

### Predicted Functions of Microbial Communities in Ileum

We utilized the PICRUSt algorithm to analyze KEGG pathways for predicting functions of intestinal microbiota related to nutrient metabolism. Four pathways associated with nutrient metabolism were analyzed including mineral absorption, carbohydrate digestion and absorption, butanoate metabolism, and starch and sucrose metabolism (Table 4). On day 21, the mineral absorption genes in XY group showed an increase (*p* = 0.046), whereas genes associated with carbohydrate digestion and absorption decreased in comparison with CON group (*p* = 0.034). On day 42, the decrease of number of genes related to butanoate metabolism was observed in XY group (*p* = 0.008), and the number of genes involved in starch and sucrose metabolism displayed a trend to decrease in XY group (*p* = 0.081).

**Table 4:** Prediction on metabolism of ileal bacterial communities using PICRUSt analysis.^1^

Items	CON	XY	Value of *p*
*day 21*			
Mineral absorption	50.67 ± 3.89	435.30 ± 26.60	0.046
Carbohydrate digestion and absorption	3929.7 ± 263.4	1330.0 ± 184.9	0.034
Butanoate metabolism	104658 ± 1374	112353 ± 2842	0.693
Starch and sucrose metabolism	137024 ± 1714	118300 ± 2605	0.185
*day 42*			
Mineral absorption	4.00 ± 0.08	73.33 ± 15.10	0.011
Carbohydrate digestion and absorption	2694.0 ± 123.5	719.7 ± 83.8	0.100
Butanoate metabolism	89377 ± 1545	58082 ± 1323	0.008
Starch and sucrose metabolism	118128 ± 3171	92262 ± 1704	0.081

1 CON is AT-xynA un-supplemented group; XY is 4,000 U/kg AT-xynA supplemented group.

Values are expressed as means ± SEM, *n* = 6.

### Acetate and Lactate Concentrations in Ileal Digesta

The lactate and acetate content in the ileal digesta of broilers are given in Table 5. Ileal samples of broilers from XY group showed a lower concentration of acetate than broilers fed CON diets on day 21 (*p* = 0.039), but no differences in acetate content were found between the two groups on day 42. No differences in lactate concentrations were observed between two treatments.

**Table 5:** Effects of AT-xynA on lactate and acetate concentrations in ileal digesta of broilers (mg/kg).^1^

Items	CON	XY	Value of *p*
*day 21*			
Lactate	1029.20 ± 49.88	1174.70 ± 155.60	0.423
Acetate	531.40 ± 20.66	311.10 ± 32.29	0.039
*day 42*			
Lactate	1992.60 ± 121.30	3054.20 ± 180.20	0.293
Acetate	424.70 ± 24.35	595.40 ± 48.20	0.411

1 CON is AT-xynA un-supplemented group; XY is 4,000 U/kg AT-xynA supplemented group.

Values are expressed as means ± SEM, *n* = 6.

### Ileal Immune Factors and Antioxidant Activities

**Figure 6:**
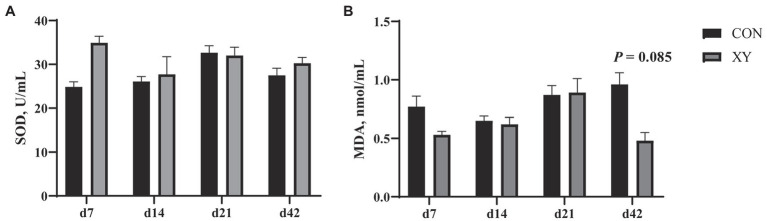
Effects of AT-xynA on antioxidant capacities in the ileum of broilers. **(A)** SOD, superoxide dismutase; **(B)** MDA, malondialdehyde concentrations in the ileum of broilers. CON is AT-xynA un-supplemented group; XY is 4,000 U/kg AT-xynA supplemented group.

We also analyzed immune factors and antioxidant activities in the ileum of broilers (Figure 6; Table 6). On day 42, the content of MDA displayed a decreasing trend in XY group (*p* = 0.085). In addition, there were no significant differences in the concentration of SOD between CON and XY groups. The concentrations of IL-1β, IL-6, and TNF-α were profoundly modulated in the ileum of broilers. In ileal tissues from AT-xynA-fed broilers, the content of IL-1β showed a trend to decrease on day 7 (*p* = 0.053). On day 14, IL-6 level significantly decreased in the ileum of broilers from XY group (*p* = 0.015), and the content of TNF-α (*p* = 0.076) and IL-1β (*p* = 0.056) tended to decrease in broilers fed with AT-xynA. On day 21, no differences were observed in IL-1β, IL-6, and TNF-α content in the ileum of broilers. On day 42, the ileal tissues of AT-xynA-fed broilers were marked by downregulation of IL-1β level (*p* = 0.047), and the content of IL-6 showed a trend to decrease (*p* = 0.067).

**Table 6:** Effects of AT-xynA on ileal immune function of broilers.^1^

Items	CON	XY	Value of *p*
*day 7*			
Interleukin-1β, pg./mg	30.94 ± 2.02	23.37 ± 1.91	0.053
Interleukin-6, ng/g	11.25 ± 1.40	9.09 ± 0.19	0.262
Interleukin-10, pg./mg	3.42 ± 0.23	3.85 ± 0.63	0.553
Tumor necrosis factor-α, ng/g	34.47 ± 3.85	29.90 ± 4.17	0.466
Interferon-γ, pg./mg	3.32 ± 0.11	3.30 ± 0.58	0.978
*day 14*			
Interleukin-1β, pg./mg	29.24 ± 4.05	18.16 ± 0.90	0.056
Interleukin-6, ng/g	14.57 ± 1.22	9.27 ± 0.45	0.015
Interleukin-10, pg./mg	4.23 ± 0.56	3.17 ± 0.25	0.155
Tumor necrosis factor-α, ng/g	40.38 ± 5.32	27.15 ± 1.57	0.076
Interferon-γ, pg./mg	4.54 ± 0.70	3.72 ± 0.24	0.329
*day 21*			
Interleukin-1β, pg./mg	42.54 ± 5.60	30.27 ± 1.92	0.107
Interleukin-6, ng/g	13.33 ± 2.59	9.28 ± 0.19	0.258
Interleukin-10, pg./mg	4.28 ± 0.29	4.63 ± 1.32	0.811
Tumor necrosis factor-α, ng/g	37.87 ± 2.79	36.97 ± 2.63	0.938
Interferon-γ, pg./mg	3.96 ± 0.22	4.31 ± 0.97	0.740
*day 42*			
Interleukin-1β, pg./mg	43.17 ± 3.51	29.37 ± 3.38	0.047
Interleukin-6, ng/g	12.31 ± 1.07	8.62 ± 1.03	0.067
Interleukin-10, pg./mg	4.09 ± 0.86	4.57 ± 1.13	0.753
Tumor necrosis factor-α, ng/g	49.41 ± 1.10	25.32 ± 3.58	0.108
Interferon-γ, pg./mg	3.87 ± 0.84	4.33 ± 0.83	0.716

1 CON is AT-xynA un-supplemented group; XY is 4,000 U/kg AT-xynA supplemented group.

Values are expressed as means ± SEM, *n* = 6.

### Ileal Barrier Functions

The results showed *ZO-1* and *occludin* gene expression levels in the ileum of broilers (Table 7). However, no significant differences were observed on *ZO-1* and *occludin* mRNA expression levels between two groups.

**Table 7:** Effects of AT-xynA on relative mRNA expression of ileal tight junction genes in broilers.^1^

Items	CON	XY	Value of *p*
*day 7*			
*ZO-1*	1.00 ± 0.18	0.80 ± 0.01	0.584
Occludin	1.00 ± 0.17	0.74 ± 0.06	0.458
*day 14*			
*ZO-1*	1.00 ± 0.13	1.41 ± 0.14	0.169
Occludin	1.00 ± 0.14	1.40 ± 0.11	0.162
*day 21*			
*ZO-1*	1.00 ± 0.14	1.02 ± 0.12	0.881
Occludin	1.00 ± 0.12	1.00 ± 0.09	0.937
*day 42*			
*ZO-1*	1.00 ± 0.09	1.11 ± 0.05	0.323
Occludin	1.00 ± 0.06	1.21 ± 0.14	0.438

1 CON is AT-xynA un-supplemented group; XY is 4,000 U/kg AT-xynA supplemented group.

Values are expressed as means ± SEM, *n* = 6. The values were calculated relative to the expression of β-actin with formula 2^−ΔΔCt^. *ZO-1*, zonula occludens-1.

### Ileal Endocrine Peptides

The effects of AT-xynA in wheat-based diets on endocrine peptide content are presented in Table 8. These results showed that concentrations of GLP-1 (*p* = 0.098) and IGF-1 (*p* = 0.03) were positively affected by xylanase supplementation on day 7. On day 21, IGF-1 concentration in the ileum of broilers fed with diets containing AT-xynA showed a trend to increase compared with CON group (*p* = 0.075).

**Table 8:** Effects of AT-xynA on ileal endocrine peptide concentrations of broilers.^1^

Items	CON	XY	Value of *p*
*day 7*			
Epidermal growth factor, pg./mg	23.45 ± 3.02	18.10 ± 0.26	0.217
Glucagon-like peptide-1, pmol/g	0.69 ± 0.04	0.89 ± 0.08	0.098
Glucagon-like peptide-2, pg./mg	200.70 ± 26.26	156.50 ± 7.41	0.181
Insulin-like growth factor-1, ng/mg	17.43 ± 1.36	21.95 ± 1.53	0.093
*day 14*			
Epidermal growth factor, pg./mg	21.37 ± 2.27	18.66 ± 1.98	0.420
Glucagon-like peptide-1, pmol/g	0.71 ± 0.02	0.99 ± 0.12	0.141
Glucagon-like peptide-2, pg./mg	153.20 ± 4.77	153.20 ± 22.33	1.000
Insulin-like growth factor-1, ng/mg	17.17 ± 0.19	22.39 ± 2.34	0.157
*day 21*			
Epidermal growth factor, pg./mg	25.34 ± 1.36	24.57 ± 7.26	0.926
Glucagon-like peptide-1, pmol/g	0.73 ± 0.02	1.12 ± 0.15	0.118
Glucagon-like peptide-2, pg./mg	201.70 ± 12.91	213.10 ± 12.73	0.843
Insulin-like growth factor-1, ng/mg	16.87 ± 1.45	28.68 ± 4.72	0.075
*day 42*			
Epidermal growth factor, pg./mg	21.21 ± 7.87	24.53 ± 2.00	0.703
Glucagon-like peptide-1, pmol/g	1.08 ± 0.34	0.88 ± 0.09	0.600
Glucagon-like peptide-2, pg./mg	181.70 ± 6.71	194.70 ± 4.87	0.853
Insulin-like growth factor-1, ng/mg	21.17 ± 7.84	22.24 ± 1.71	0.905

1 CON is AT-xynA un-supplemented group; XY is 4,000 U/kg AT-xynA supplemented group.

Values are expressed as means ± SEM, *n* = 6.

### Correlations Between Ileal Microbiota, Lactate and Acetate Levels, Morphology, and Immune Factors

**Figure 7:**
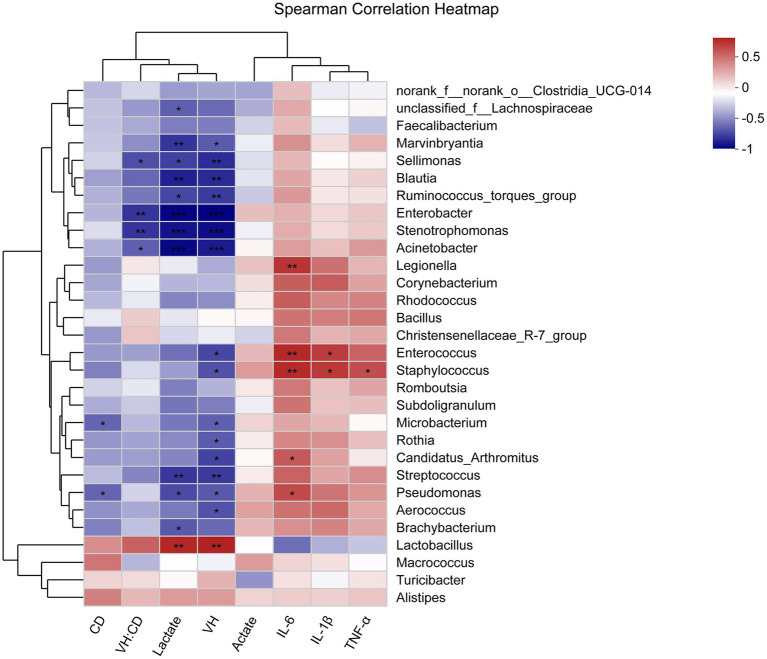
Heatmap of spearman’s correlation between ileal microbiota, metabolites, morphology, and immune factors. The correlation was analyzed based on the relative abundance of 30 phylotypes at genus level. The blue suggests a negative correlation, and the red suggests a positive correlation. The “*” indicates 0.01< *p* ≤ 0.05, “**” indicates 0.001< *p* ≤ 0.01, and “***” indicates *p* ≤ 0.001.

The correlations between ileal microbiota, metabolites, morphology, and cytokine levels were evaluated by the spearman correlation analysis (Figure 7). The concentration of lactate was positively associated with the abundance of *Lactobacillus* and negatively associated with the abundance of *Pseudomonas*, *Brachybacterium*, *unclassified_f__Lachnospiraceae*, *Enterobacter*, *Streptococcus*, *Marvinbryantia*, *Sellimonas*, *Blautia*, *Ruminococcus_torques_group*, *Stenotrophomonas*, and *Acinetobacter* (*p* < 0.05). The intestinal development (higher VH, lower CD, and higher VH/CD suggesting greater intestinal development) exhibited a positive correlation with the abundance of *Lactobacillus* and a negative correlation with *Enterococcus*, *Staphylococcus*, *Pseudomonas*, *Aerococcus*, *Microbacterium*, *Rothia*, *Candidatus_Arthromitus*, *Enterobacter*, *Streptococcus*, *Marvinbryantia*, *Sellimonas*, *Blautia*, *Ruminococcus_torques_group*, *Stenotrophomonas*, and *Acinetobacter* (*p* < 0.05). The immune functions (lower levels of IL-1β, IL-6, and TNF-α indicating greater immune functions) displayed a negative correlation with the abundance of *Legionella*, *Enterococcus*, *Staphylococcus*, *Pseudomonas*, and *Candidatus_Arthromitus* (*p* < 0.05).

## Discussion

In the current study, AT-xynA supplementation could improve broilers growth performance, and this observation was anticipated and consistent with published reports. González-Ortiz et al. (2016) demonstrated that supplementing xylanase to wheat-based diets could numerically increase body weight gain (BWG) and FCR of broilers. Zhang et al. (2014) also showed improved FCR and BWG of broilers fed wheat-based diets supplemented xylanases. However, positive effects of AT-xynA on growth performance of broilers were not observed until the third week of age after hatch, indicated by increased ADG from days 14–21 and days 21–42. It has demonstrated that the development of digestive system of chicks occurs rapidly in the first few days after hatching, and nutrients were utilized preferentially for the gastrointestinal tract development of chicks relative to BWG (Lji et al., 2001).

The process of early intestinal growth and development involves changes in intestinal morphology, and the enhanced intestinal growth could efficiently improve absorption ability and nutrient utilization, and may further benefit to the subsequent growth performance (Dai et al., 2020). We found that the addition of AT-xynA could improve ileal VH, and the improvement in VH might be related to the greater absorption surface and faster epithelial cells turnover, suggesting improvement of nutrient absorption capacity in the intestine (Li et al., 2019). Similarity, Wu et al. (2004a) suggested that the addition of xylanase to wheat-based diets increased VH in the ileum of broilers on day 21. Luo et al. (2009) also reported that xylanase could increase ileal VH and VH/CD ratio in broilers fed wheat-based diets. Dietary NSPs could negatively influence epithelial cell turnover of the intestine, but degradation of xylans might be able to alleviate these negative effects on intestinal morphology in broilers (Lee et al., 2020). However, some reports showed that xylanase supplementation had no effects on VH, CD, and VH/CD ratio of the ileum in broilers, and we hypothesized that the type of wheat, proportion of dietary wheat, characteristics of xylanase, and age of broilers might be the reason why broilers showed different responses to xylanase supplementation (Wu et al., 2004b). In fact, no significant differences in ileal morphology of broilers were found on day 42 in the current study. This may be explained by the development of intestine in broilers, and the morphological maturation of the small intestine occurs during the first week (Lji et al., 2001). Thus, young broilers might be particularly sensitive to NSP, and benefits of xylanase in young broilers are greater.

In addition to direct effects of gut physiology and morphology by viscous NSPs, the negative effects of soluble NSPs on broilers performance appear to be indirectly associated with changes of intestinal microbiota composition. The type of diets and dietary additives has been reported to be able to affect the composition of intestinal microbiota in broilers (Sudhir and Jha, 2019). The alpha diversity of broilers intestinal microbiota could reflect richness and evenness of microbial community (Wagner et al., 2018). In this study, the alpha diversity of ileal microbiota obviously decreased with xylanase supplementation. Hübener et al. (2002) demonstrated that xylanase exhibited a reduction in the total microbial load in terms of bacteria counts in the small intestine. The broilers fed wheat-based diets resulted in the increase of digesta viscosity, and the viscous digesta could reduce intestinal mobility and decrease oxygen tension, thus stimulated the colonization of certain bacteria in the ileum, particularly pathogenic microbiota (Hübener et al., 2002). Moreover, undigested protein and starch in the small intestine might result in the bacterial overgrowth (Bedford, 2018). Supplementing xylanase to wheat-based diets could decrease the amount of nutrients which are available as substrates utilized by bacteria in the ileum, thus reduced the number of bacteria in the small intestine (Choct et al., 1999; Apajalahti et al., 2004).

At the phylum level, similar to previous reports, Firmicutes was dominant in the ileum of broilers (Rychlik, 2020). On days 21 and 42, the abundance of Proteobacteria was lower in broilers supplemented xylanase. It has been demonstrated that many human pathogens, such as *Escherichia*, *Campylobacter*, *Salmonella*, *Helicobacter*, and *Pseudomonas*, were found in Proteobacteria phylum (Rizzatti et al., 2017). Thus, the enhanced abundance of Proteobacteria might be a microbial signature for diseases. The current study demonstrated that the abundance of Lactobacillaceae (belonging mainly to the genus *Lactobacillus*) in the ileum was promoted by xylanase supplementation. Engberg et al. (2004) also reported that xylanase supplementation could increase the abundance of *Lactobacillus* in the ileum of broilers fed wheat-based diets. *Lactobacillus* spp. is beneficial to the gut health of broilers due to probiotic effects; it could lead to the competitive exclusion of the pathogenic bacteria (Gao et al., 2017). In the current study, we found that *Lactobacillus* enrichment was positively correlated with lactate concentration and mucosal VH in the ileum of broilers. The previous study also reported that the abundance of *Lactobacillus* was positively related to ileal VH in layer chicks (Dai et al., 2020).

On day 21, the abundance of genus *Rothia*, *Glutamicibacter*, and *Micrococcus* (belonging to the family Micrococcaceae) was higher in broilers fed wheat-based diets un-supplemented xylanase. The genus *Rothia* has been reported to be able to result in serious infections, particularly in immunocompromised hosts (Ramanan et al., 2014). The current study also showed that *Rothia* was negatively associated with the intestinal development. However, the knowledge about metabolism and role of genus *Glutamicibacter*, *Micrococcus*, and family Micrococcaceae was limited. Higher abundance of genus *Microbacterium, Enterobacter*, *Staphylococcus*, and *Pseudomonas* in the ileum was also observed in broilers fed CON diets. Strains belonging to the genus *Microbacterium* (within the family Microbacteriaceae), genus *Enterobacter* (within the family Enterobacteriaceae), genus *Staphylococcus* (within the family Staphylococcaceae), and genus *Pseudomonas* (within the family Pseudomonadaceae) have recently been identified as pathogenic bacteria and might be related to a wide range of infections (Funke et al., 1997; Hotterbeekx et al., 2016). We also found that the abundance of *Enterobacter*, *Staphylococcus*, and *Pseudomonas* was negatively correlated with lactate concentration, development of intestinal morphology, and ileal immune function. On day 42, remarkable increase in the abundance of pathogenic bacteria, such as family Moraxellaceae, Nocardiaceae, Burkholderiaceae, Staphylococcaceae, genus *Streptococcus*, and *Enterococcus*, was shown in broilers from CON group; these bacteria contain a few animal pathogens which might result in a wide variety of infections (Arana et al., 2001; Chadfield et al., 2005; Neveling et al., 2017; Chen et al., 2019; DiCenzo et al., 2019). The higher abundance of pathogenic bacteria might explain poor performance of broilers from CON group. In addition, higher abundance of genus *Frisingicoccus*, *Eubacterium_hallii_group*, and *Ruminococcus* was observed in xylanase un-supplemented broilers on day 21, and higher abundance of Peptococcaceae and Bacillaceae was shown in broilers from CON group on day 42. These bacteria belong to phylum Firmicutes; however, this phylum is thought to contribute to the production of SCFA (Dai et al., 2021). Supplementing xylanase could also decrease the abundance of family Propionibacteriaceae on day 21, and bacteria belonging to family Propionibacteriaceae could also promote the production of SCFA. Choct et al. (1996) have reported that supplementing soluble NSPs to broilers diets could elevate fermentation in the small intestine. The increase in the abundance of SCFA-producing bacteria might be related to the alleviation of excess fermentation due to xylanase.

In PICRUSt function analysis, the ileal microbiota of broilers supplemented xylanases showed an increase in mineral absorption pathway. This result demonstrated that broilers from XY group could have a higher capacity to utilize minerals. However, bacterial carbohydrate digestion and absorption, butanoate metabolism, and starch and sucrose metabolism pathways were higher in CON group. The upregulation in these pathways might be due to high NSP content present in broilers provided wheat-based diets un-supplemented xylanase. The nutrients in the ileum of broilers fed diets un-supplemented enzymes were exposed; however, nutrients had little chance to contact digestive enzymes in the small intestine due to high digesta viscosity, and undigested nutrients were fermented by microbiome to produce SCFA. In this study, we also have found that bacterial fatty acid production increased in the ileum of broilers from CON group. Supplementing NSP-degrading enzymes to diets also has been reported to improve ileal starch digestibility of broilers (Choct et al., 1996). Given that nutrient digestion and absorption capacity significantly increased in XY group, lower carbohydrate digestion and absorption, and starch and sucrose metabolism pathways in XY group were likely due to less nutrients present in ileal content for bacteria to utilize and hence a compensatory upregulation in metabolic pathways.

Lower concentration of acetate was observed in broilers fed xylanase in the current study. Choct et al. (1996) demonstrated that broilers fed high NSP diets showed higher concentrations of SCFA in the ileum compared with broilers fed enzyme-supplemented diets. Józefiak et al. (2007) also reported that adding xylanase to broilers rye-based diets could decrease SCFA concentrations in the ileum. The addition of xylanases could effectively reduce the amount of substrates available for the colonization of fermentative bacteria, and the reduction in microbial fermentation could decrease the production of SCFA and alleviate excess fermentation in the ileum (Choct et al., 1999). Higher abundance of *Lactobacillus* was observed in broilers fed xylanase; however, no differences in lactate concentrations were observed in broilers from the two groups. We hypothesized that lactate produced by *Lactobacillus* was absorbed in the intestine or used as a substrate for lactate-utilizing bacteria.

It has been reported that the immune system could be affected by providing feed additives to diets that could influence the composition of intestinal microbiota (Liu et al., 2012; Schokker et al., 2017). We found the reduction in IL-1β and TNF-α concentrations of ileal tissues in broilers from XY group. IL-1β and TNF-α are important proinflammatory cytokines that produced and released by macrophages, and they are considered to regulate pathological responses that occur in inflammatory conditions (Jensen et al., 2020). Zhang et al. (2018) demonstrated that supplementing xylanases and fermented polysaccharide of *Hericium caputmedusae* (FPHC) increased the anti-inflammatory capacity of broilers by improving serum IL-1α and IL-10, and decreasing IL-1β and TNF-α concentrations. Wang et al. (2021) reported that the exogenous enzyme complex (primarily contained 3,200 U/kg β-glucanase and 6,225 U/kg xylanase) could significantly increase the levels of IL-6 and IL-10, and decrease IL-1β and TNF-α levels in the jejunum of broilers. Reduction in the serum TNF-α content in nursey pigs fed diets with corn distiller’s dried grains was also observed (Chen et al., 2020). Similar to IL-1β, IL-6 is another pro-inflammatory cytokine, and it responses to immune stimulation (Umare et al., 2014). It has been widely accepted that nutrition could impact immune function, and deficiency in nutrients could negatively affect normal immune responses (Wu et al., 2018). Thus, the addition of xylanases could promote the digestion and absorption of nutrients by degrading arabinoxylans, which in turn may contribute to the improvement of immune function. Moreover, extensive interactions occur between intestinal microbiota and immune system of the host (Sudhir and Jha, 2019). We have observed that the addition of xylanase stimulated the colonization of beneficial bacteria and reduced the abundance of harmful bacteria in the intestine of broilers. Therefore, the results suggested that xylanase might modulate immune responses in broilers by regulation of intestinal microbiome that positively affects nutrient digestion and utilization along with positively affects intestinal immunity. At the same time, we observed that effects of xylanase on immunity function were greater at young age. After hatch, the development of immune system and colonization of intestinal microbiota occur rapidly; in addition, the microbiota colonization in broilers is essential for this development (Bar-Shira et al., 2003). Thus, we hypothesized that older broilers had a greater capacity to deal with negative effects of NSP due to mature gut microbiota and immune system.

Oxidative stress is essential to the intestinal health of broilers; MDA as one of the essential toxic lipid peroxides could cause damage to the membrane structure and function (Gawe et al., 2004). In this study, these results suggested that xylanase supplementation could enhance the antioxidant capacity of broilers. Zhang et al. (2018) has demonstrated that xylanase and FPHC treatment reduced the level of serum MDA in broilers. Wang et al. (2021) reported that the exogenous enzyme complex (primarily contained 3,200 U/kg β-glucanase and 6,225 U/kg xylanase) could improve the activities of T-AOC, SOD, and GSH-Px in the jejunal mucosa of broilers. The application of xylanase could also effectively reduce the content of MDA in the mucosa of jejunum in newly weaned piglets (Duarte et al., 2019). Petry et al. (2020) reported that xylanase supplementation improved the antioxidant capacity in the ileum of growing pigs provided insoluble corn-based fiber. However, it is still unclear how xylanase could reduce oxidative stress and improve antioxidant capacity of broilers. Duarte et al. (2019) thought that negative effects of NSPs toward digesta could influence molecules in the intestinal cell wall, and this may be the reason why xylanase could decrease oxidative stress.

The intestinal epithelial barrier function is the first line of defense against intruders of the body, and intestinal barrier dysfunction might result in the loss of integrity of the epithelium and an increase risk of gastrointestinal diseases (Camilleri et al., 2012). The barrier contains several unique proteins; occludin and *ZO-1* are two essential components in the modulation of barrier function of the intestine (Fanning et al., 1998). In the current study, no significant differences were observed in *occludin* and *ZO-1* mRNA expression levels in the ileum of broilers from two groups. However, Petry et al. (2020) suggested that supplementing xylanase could upregulate *claudin-4* and *occludin* mRNA expression level in the ileum of growing pigs fed insoluble corn-based fiber. Tiwari et al. (2018) also reported that xylanase supplementation increased *claudin*, *occludin*, and *ZO-1* mRNA expression in the jejunum of nursey pigs fed corn distiller’s dried grain with soluble based diets. In this study, no differences in the intestinal barrier function were observed, and one potential speculation could be that the content of NSP which caused barrier function damage was insufficient.

L-cells as important component of the gut-brain axis are primarily located in the jejunum and ileum of chickens; the endocrine peptides secreted from L-cells are essential to the digestion of nutrients and development of the intestine (Lim et al., 2009). Higher levels of GLP-1 and IGF-1 were observed in the ileum of broilers from XY group. Glucagon-like peptide-1 has been demonstrated to play a crucial pole in gastric emptying reduction and small intestine growth rate (Lim et al., 2009). As a result of the increase of GLP-1 concentration, the nutrients could be more efficiently digested and absorbed. Gao et al. (2007) also reported that supplementing xylanase could increase blood IGF-1 content in 21-day-old cockerels. Insulin-like growth factor-1 also benefits to the growth rate and energy metabolism; this endocrine peptide is beneficial to the growth and function of almost every organ (Wang et al., 2017). Insulin-like growth factor-1 has been reported to increase VH in rats (Mantell et al., 1995); thus, enhanced IGF-1 content in broilers supplemented xylanase might partly explain why improvement in ileal morphology was observed. However, we only observed that the content of IGF-1 and GLP-1 in XY group was higher than that in CON group on days 7 and 21. The intestine microbiota of older broilers might be more mature and stable, the ability of microbiota to degrade arabinoxylans grew, and thus, xylanase supplementation had a weak positive effect on intestinal health (Bautil et al., 2019). This might suggest that the effects of xylanase are more pronounced in young broilers due to their low digestive capacity to wheat-based diets.

## Conclusion

Xylanase supplementation to broiler wheat-based diets had beneficial effects on ileal microbiota composition, which was reflected in enhanced relative abundance of *Lactobacillus*, and reduced relative abundance of potentially pathogenic bacteria. Xylanase supplementation decreased the abundance of SCFA-producing bacteria and acetate concentration in the ileum; this reduction suggested that xylanase could alleviate excess fermentation of bacteria. In addition, AT-xynA could improve ileal morphology and decrease content of proinflammatory cytokines, which were associated with alterations in ileal microbiota induced by xylanase supplementation. The positive effects of xylanase on the intestinal health were more pronounced in young broilers, indicating an adaptive effect was exhibited as broilers aged.

## Data Availability Statement

The datasets presented in this study can be found in online repositories. The names of the repository/repositories and accession number(s) can be found at: https://www.ncbi.nlm.nih.gov/, PRJNA725811.

## Ethics Statement

The animal study was reviewed and approved by The Laboratory Animal Welfare and Animal Experimental Ethical Inspection Committee of China Agricultural University (Beijing, China).

## Author Contributions

JW and XP conceived and designed the experiments. JW, SL, and JM performed the animal experiments. JW analyzed the data and wrote the manuscript. XP supervised and provided continuous guidance for the experiments. All authors have discussed the results and reviewed the manuscript.

### Conflict of Interest

The authors declare that the research was conducted in the absence of any commercial or financial relationships that could be construed as a potential conflict of interest.
